# Accidental Bowel Transgression/Close Proximity During Percutaneous Microwave Ablation of Liver Tumors: A Retrospective Case Series

**DOI:** 10.3390/jcm15083171

**Published:** 2026-04-21

**Authors:** Krish Vennam, George Ashji, Ashwani Kumar Sharma

**Affiliations:** 1 Strong Memorial Hospital, University of Rochester, Rochester, NY 14642, USA; krish_vennam@urmc.rochester.edu; 2 Department of Biochemistry, University of Rochester, Rochester, NY 14642, USA; 3 Department of Imaging Sciences, University of Rochester, Rochester, NY 14642, USA

**Keywords:** HCC, ablation, gut transgression, bowel injury

## Abstract

**Aim**: Percutaneous liver ablation is a challenging procedure and operator-dependent. During the time when transarterial liver oncological therapies are favored over percutaneous liver ablation, we discuss the challenges of liver ablation with bowel interposition within the needle tract. **Materials and Methods**: In this IRB-approved retrospective review, we analyzed 481 cases of percutaneous microwave ablation performed between 2012 and 2025 using the NeuWave microwave ablation system with 15 or 20 mm probes under non-contrast CT guidance, with needle trajectories planned based on ultrasound. Dissection techniques were not performed, as intraprocedural ultrasound and CT assessment suggested that the ablation zone would remain confined to hepatic parenchyma. Cases of bowel transgression or close proximity were identified on post-procedural CT imaging, with a follow-up duration of 3 months performed consistently across all cases. **Results**: Three cases (0.6%) of bowel transgression or close proximity to bowel loops during needle placement were identified. There was no evidence of transmural bowel perforation or clinically significant bowel injury on clinical or radiologic follow-up. Post-procedural imaging demonstrated no free intraperitoneal air or fluid collections. **Conclusions**: In cases where the ablation zone is confined to hepatic parenchyma, bowel proximity to or inadvertent traversal by the cooled antenna shaft may not result in clinically significant injury and can be managed conservatively in selected patients.

## 1. Introduction

Percutaneous image-guided thermal ablation is an essential treatment modality for selected liver tumors. This is particularly important for patients who are not candidates for surgical resection due to tumor location, comorbidities, or limited hepatic reserve. In these settings, ablation often serves as the primary curative-intent option and cannot be readily replaced by alternative therapies such as surgery or transarterial treatments. Data demonstrate that thermal ablation provides comparable outcomes and overall survival to surgical resection for small tumors, particularly those ≤3 cm. It also offers reduced morbidity in appropriately selected patients [1,2]. These findings emphasize the importance of understanding the safety of ablation in anatomically challenging cases.

A major limitation of percutaneous liver ablation is the proximity of tumors to adjacent non-target structures, particularly bowel loops. When bowel is located near the planned ablation zone, there is concern for thermal injury and perforation, and bowel proximity has traditionally been considered a high-risk scenario. Although gastrointestinal injury is relatively uncommon, occurring in approximately 0.11% of cases, it remains one of the most serious complications of thermal ablation [3]. In clinical practice, these situations require careful procedural planning.

Image guidance plays a critical role in navigating these complex cases. Ultrasound and CT are commonly used for procedural guidance, and newer techniques such as transcatheter CT hepatic arteriography (CTHA) can further improve tumor visualization, navigation, and assessment of surrounding anatomy [4,5]. Despite these advances, adequate separation between the liver and bowel cannot always be achieved. As a result, adjunctive techniques such as hydrodissection or pneumodissection are often employed to increase the distance between the liver and bowel, particularly when a safe trajectory cannot be achieved with positioning alone. Additional strategies, including angiographic balloon interposition and alternative access routes, such as intercostal or subcostal approaches, may also be used to reduce the risk of injury [6,7]. Despite these measures, close proximity to the bowel or inadvertent traversal may still occur due to limited windows of access and challenging anatomy.

While thermal injury is well described when bowel is included within the active ablation zone, the risk associated with bowel proximity to the cooled antenna shaft is less clearly understood. Injury from the active ablation tip results from exposure to cytotoxic temperatures, leading to coagulative necrosis, whereas the antenna shaft is internally cooled and does not generate sustained cytotoxic temperatures along its length [8,9,10]. Despite this theoretical difference, there is limited clinical data describing outcomes when the bowel is in direct contact with, or very close to, the antenna shaft.

Alternative therapies such as yttrium-90 (Y90) radioembolization may be considered in anatomically challenging cases, as they avoid direct needle-related injury to adjacent structures. However, these treatments are not always equivalent to curative-intent ablation for small, localized tumors and are typically selected based on individual tumor and patient characteristics [11,12,13]. Given these considerations, there are currently no standardized guidelines defining safe distances between the bowel and the antenna shaft, which creates uncertainty for operators when managing these scenarios.

This study aims to evaluate clinical outcomes in cases of bowel transgression or near proximity during percutaneous liver ablation, with a focus on whether such situations are associated with clinically significant bowel injury.

## 2. Materials and Methods

This study was a single-center retrospective review of patients who underwent percutaneous microwave ablation of liver tumors between January 2012 and December 2025. Institutional Review Board (IRB) approval was obtained (IRB #STUDY00001919, approved on 8 October 2018), with waiver of informed consent due to the retrospective nature of the study. Patients were identified through procedural records of percutaneous liver ablation. Cases were included if bowel transgression or near proximity to the bowel was identified on post-procedural imaging. No additional demographic-based inclusion or exclusion criteria were applied, as the study was focused on technical and procedural outcomes.

Bowel transgression was defined as direct passage of the antenna shaft across a bowel loop as identified on post-procedural imaging, while near proximity was defined as immediate adjacency of the antenna shaft to the bowel without a visible intervening fat plane. All cases were assessed using post-procedural CT imaging, with evaluation for associated findings including intraperitoneal free air, fluid collections, and bowel wall abnormalities.

All procedures were performed using a microwave ablation system (Neuwave Medical, Madison, WI, USA) with 15 mm or 20 mm antenna probes. The antenna utilizes an internally cooled shaft system. Ablations were performed at 65 W for approximately 10 min, depending on lesion characteristics. Procedures were performed under combined ultrasound and non-contrast CT guidance. Needle trajectories were planned primarily using ultrasound to optimize angulation and access, particularly in cases with limited procedural windows, with CT used for confirmation of antenna position and post-procedural assessment. No hydrodissection or pneumodissection techniques were performed, as intraprocedural ultrasound with CT confirmation demonstrated a planned trajectory without significant bowel involvement.

All patients underwent routine post-procedural imaging and clinical follow-up at 3 months, with continued longitudinal follow-up thereafter. Outcomes assessed included evidence of bowel injury, need for intervention, and development of delayed complications.

## 3. Results

**Figure 1:**
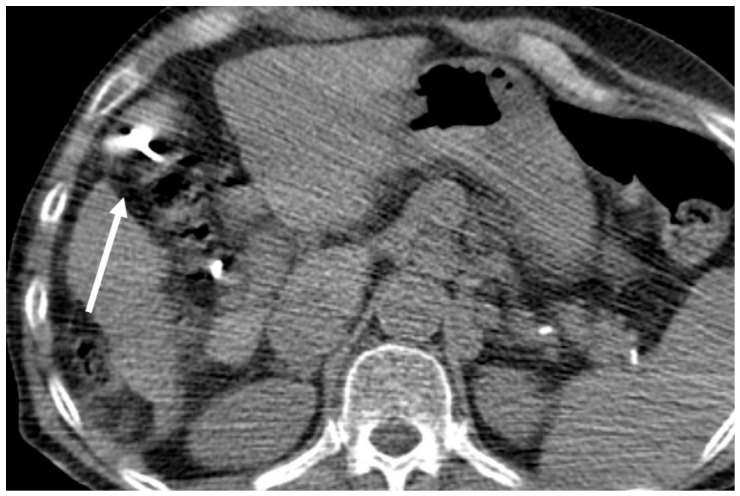
Right: Axial CT scan shows the accidental transgression/close proximity of the ablation needle through the bowel loops (arrow).

**Figure 2:**
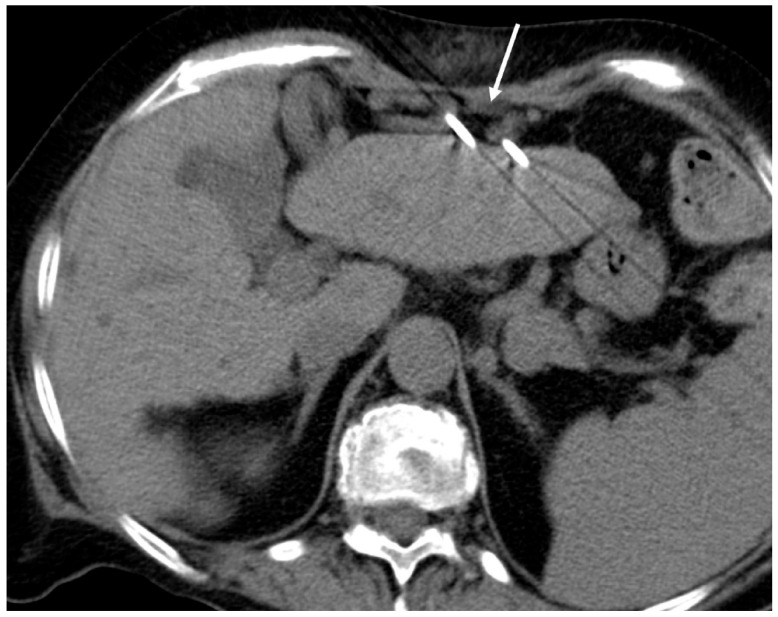
Left: Axial CT scan shows the accidental transgression/close proximity of the ablation needle through the bowel loops (arrow).

We found 3 cases where there was accidental transgression of the needle through the bowel wall during needle placement (Figure 1 and Figure 2). Among the 481 percutaneous microwave liver ablation procedures performed over the study period (January 2012 to December 2025), this corresponded to an observed incidence of 0.6%. In the overall cohort, most patients had early-stage disease with isolated hepatic lesions. Barcelona Clinic Liver Cancer staging was limited to early stages, with approximately 90% of patients classified as stage A and the remainder as stage 0. Functional status was generally well preserved, with ECOG performance status of 10% ECOG 0, 36–40% ECOG 1, 48% ECOG 2, and 2% ECOG 3. All three cases of bowel transgression occurred in patients with BCLC stage A disease and ECOG performance status 1, consistent with the predominant characteristics of the overall cohort. Two tumors were located in the right hepatic lobe and one in the left, with involved small bowel loops adjacent to the hepatic lobes and no colonic involvement. No additional cases of clinically significant bowel perforation or delayed gastrointestinal complications were identified within the remaining cohort. Given the technical focus of this study, detailed patient demographic data were not included, as the analysis was centered on procedural characteristics and outcomes.

With the following three cases that received microwave ablation, needle trajectories were planned based on ultrasound, and initial intraprocedural assessment suggested an adequate access path without significant bowel involvement. As a result, hydrodissection or pneumodissection was not performed. Post-procedural CT imaging, however, demonstrated inadvertent bowel transgression or immediate proximity of the antenna shaft to adjacent bowel loops.

In each of the three cases, several imaging and clinical findings supported conservative management. Immediate post-procedural imaging confirmed that the active tip of the microwave antenna remained entirely within the hepatic lesion and that the ablation zone did not involve the bowel. There was no bowel wall thickening, loss of enhancement, surrounding inflammatory change, pneumoperitoneum, or adjacent fluid collection. Clinically, patients remained hemodynamically stable without abdominal pain, peritoneal signs, fever, or leukocytosis.

At the end of the procedure, the needles were removed. Only the needle tract through the liver parenchyma was cauterized under ultrasound guidance. No cauterization was applied along the extrahepatic or bowel-transgressed portion of the shaft to avoid potential thermal injury outside the liver. Patients were discharged the same day on oral antibiotics (cephalexin 500 mg three times daily and metronidazole 500 mg three times daily for 10 days) and all recovered without complication. A three-month follow-up was performed consistently across all patients, with continued longitudinal follow-up thereafter.

During post-procedural monitoring, patients remained hemodynamically stable, and no patient required hospital admission, surgical consultation, or escalation of care related to the event. There was no abscess formation, no fistula development, and no delayed perforation. In addition, no patient required delayed readmission or intervention related to the mechanical traversal.

## 4. Discussion

Bowel injury during percutaneous liver ablation is uncommon, with reported rates of adjacent organ injury below 1% [3]. However, it remains a feared complication of thermal therapy due to the potential for perforation, peritonitis, abscess formation, need for surgical intervention, and even death. For this reason, bowel interposition along the ablation trajectory is traditionally considered a contraindication, and careful efforts are made to establish a safe access path prior to energy delivery.

It is important to emphasize that intentional traversal of the bowel should always be avoided during percutaneous liver ablation. Operators should make every effort to select a trajectory that avoids bowel loops, including the use of patient repositioning, hydrodissection, or alternative access routes when necessary. The cases presented here represent rare instances of inadvertent mechanical bowel transgression or close proximity of the antenna shaft despite appropriate planning and imaging guidance.

In this cohort of 481 microwave ablation procedures, three cases of accidental bowel transgression were identified, corresponding to an incidence of 0.6%. Notably, none of these cases resulted in clinical or radiologic evidence of bowel perforation, abscess formation, fistula, or delayed gastrointestinal complication. All cases represented mechanical shaft traversal or proximity without inclusion of bowel within the active thermal field. This distinction is critical in understanding the favorable clinical outcomes observed.

Prior reports of gastrointestinal injury following thermal ablation of liver tumors have largely attributed complications to thermal injury within the ablation zone. In contrast, modern microwave ablation systems with gas-cooled antenna technology are designed to confine cytotoxic temperatures to the active tip while minimizing conductive heat propagation along the shaft. These systems circulate gas, typically carbon dioxide, to dissipate heat via the Joule–Thomson effect, allowing the shaft to remain at substantially lower temperatures during energy delivery [14]. Experimental data demonstrate that internally cooled shafts remain below 20 °C, whereas non-cooled systems may exceed 90 °C with unintended tissue coagulation along the shaft [15]. Clinical series have also reported lower complication rates with cooled-shaft systems, with major adverse event rates of approximately 3% in contemporary studies [16,17,18].

When the active ablation tip remains confined to hepatic parenchyma, and bowel is excluded from the ablation zone, the risk of thermal injury to adjacent structures is low. Despite these well-described properties, existing literature has focused on injury from bowel inclusion within the ablation zone, and to our knowledge, there are no prior studies specifically evaluating outcomes following inadvertent bowel transgression or direct shaft proximity during liver ablation.

The findings of the present study suggest that this distinction is clinically meaningful. In all cases, post-procedural imaging demonstrated no bowel wall thickening, hypoenhancement, pneumoperitoneum, or adjacent fluid collection, and patients remained clinically stable without signs of peritonitis, fever, or leukocytosis. These findings supported a conservative, non-operative approach. While a three-month follow-up was sufficient in this series, earlier imaging at approximately 1 month may be reasonable to evaluate for delayed complications such as abscess or fistula formation.

Antibiotic therapy was administered in all cases to mitigate the potential risk of bacterial translocation despite the absence of overt perforation. In clinically stable patients without concerning imaging or examination findings, this supports the use of a conservative outpatient approach with oral antibiotics and close follow-up.

It is equally important to recognize situations in which conservative management may not be appropriate. If imaging demonstrates bowel inclusion within the ablation zone or findings suggestive of bowel injury, including wall thickening, hypoenhancement, progressive pneumoperitoneum, or fluid collection, early surgical consultation should be considered. Additionally, if patients develop clinical signs of peritonitis or systemic instability, prompt surgical evaluation is warranted.

When bowel interposition precludes a safe percutaneous approach, alternative treatment strategies such as transarterial embolization, radioembolization, or surgical resection may be considered. While effective, these options carry distinct limitations, including delayed tumor response, need for multiple treatments, and differing risk profiles. Percutaneous ablation remains the preferred modality when a safe access route can be achieved, offering effective local control with minimal invasiveness.

This study has several limitations. Its retrospective, single-center design introduces potential selection and reporting bias, and the small number of events limits generalizability. Subclinical microperforation cannot be definitively excluded, as routine endoscopic evaluation was not performed. Additionally, follow-up was limited to three months, and longer-term outcomes remain unknown. Finally, these findings reflect experience with gas-cooled microwave systems and may not be generalizable to other ablation technologies.

Future studies should evaluate larger, multicenter cohorts to better define the incidence and predictors of outcomes following mechanical bowel transgression or close proximity. Further investigation into thermal gradients along cooled antenna shafts may help refine safety margins and improve procedural planning in anatomically complex cases.

## 5. Conclusions

When bowel loops are adequately prepared, and the active ablation tip remains confined to hepatic parenchyma, bowel proximity to or traversal by the cooled antenna shaft does not appear to result in clinically significant injury. In the absence of imaging or clinical evidence of bowel injury, conservative management with observation, antibiotics, and follow-up imaging may be considered in carefully selected patients.

## Data Availability

The original contributions presented in this study are included in the article. Further inquiries can be directed to the corresponding author.
